# Adaptive minirhizotron for pepper roots observation and its installation based on root system architecture traits

**DOI:** 10.1186/s13007-019-0414-z

**Published:** 2019-03-23

**Authors:** Wei Lu, Xiaochan Wang, Fengjie Wang

**Affiliations:** 1 Department of Engineering College, Nanjing Agricultural University, Nanjing, 210031 China; 2 Jiangsu Province Engineering Laboratory for Modern Facilities Agricultural Technology and Equipment, Nanjing, 210031 China

**Keywords:** Adaptive minirhizotron, Pepper roots, Root 3D reconstruction, Root analysis, Installation patterns, Root observation

## Abstract

**Background:**

Root is the principal part of plants to absorb water and nutrient, anchor the plant and affect yield and quality. Non-destructive detection of root traits is difficult to perform due to the hidden nature of the root. Therefore, improved methods to measure roots are necessary to support plant breeding, and optimization of cultivation and management. In this study, we present an adaptive minirhizotron along with installation patterns to focus on micro and local changes in multipoint of pepper roots.

**Results:**

The method is to improve minirhizotron by reducing its size to a microrhizotron (1.1 × 1.1 × 1.2 cm) and improving installation validity and rationality according to spatial distribution characteristics of *Capsicum annuum* root system. This adaptive minirhizotron could acquire root image in multipoint, and through image processing, root traits such as root length (including very fine roots or root hairs) and root width could be calculated. In order to install the microrhizotron reasonably and effectively, root system architecture (*Capsicum annuum*) was reconstructed using a three-dimensional caliper, and were quantified in circumferential distribution, vertical direction and root extension. The results showed that most lateral roots were constrained to 45° in horizontal direction to root initial position; Vertical angles were large, almost perpendicular to the root center line at initial position, and it became smaller when roots began to deepen. Root length density decreased with the increase of distance to plant center. According to *Capsicum annuum* root system traits, totally 8 installation methods were tested and verified to determine high probability of root interception. Horizontal angle 45° showed much higher interception probability than that of 90°. Vertical angle 45° has slightly higher root interception probability than that of 30°. Installation pattern horizontal angle 45° + radius 30 mm + vertical angle 45° showed the best performance in root interception with probability of 96.7%, followed by pattern horizontal angle 45° + radius 30 mm + vertical angle 30°. Comparison experiment showed that when root hair and very fine root were excluded, relative error was 12.1% between microrhizotron and soil sampling in root length, and 15.4% in root diameter. Microrhizotron was able to observe fine roots about 0.1 mm in diameter.

**Conclusion:**

A new adaptive minirhizotron has been established for nondestructive observation on local and micro changes of roots in multipoint, and its application and installation patterns has been suggested according to root architecture traits. The microrhizotron can be used to study a wide range of research questions focused on quantitative trait locus analysis, root width changes, and root hair growth.

**Electronic supplementary material:**

The online version of this article (10.1186/s13007-019-0414-z) contains supplementary material, which is available to authorized users.

## Background

Being the principal water and nutrition absorbing organ, root plays a very important role in anchor of plants, uptake of water and nutrient, and affecting crop yield and quality. The study of root morphology is the most important content in plant nutrition, plant physiology, breeding, and ecology [1, 2]. Accurate acquisition of root morphology in real time is the prerequisite and key step to gene improvement, water and fertilizer utilization efficiency, and crop quality and yield in agricultural production [3]. Due to the opacity and complexity of root growth environment, the research progress of roots is relatively slow. Traditional methods (excavation, profile, soil column, etc.) are mostly destructive or have changed the original growth environment of crops (hydroponics, fogging), and the test results are not of universality [4–6].

With the development of computer technology and nondestructive testing technology, methods such as X-ray computed tomography (X-CT), radioisotope tracing, nuclear magnetic resonance (NMR) and minirhizotron have been developed [7–9]. X-CT, radioisotope tracing, and NMR were expensive, difficult to operate, and can only be measured in a laboratory environment, so they have not been widely used currently [7, 9–13]. Minirhizotron, first described by Bates in 1937, was a kind of nondestructive method to observe roots at a particular point throughout the growing period [14–16]. Since then, a lot of researches such as forest, orchard, agroecosystem and other areas have been carried out based on application of minirhizotron [17–21]. Upchurch [22] used a low-light monochromatic camera to conduct in situ root observation through a transparent acrylic canal, which proved that this method has a linear relationship with the soil sampling method. Liao [23] used minirhizotron observation method to track and monitor the root growth dynamics of maize over a period of time, and also showed that this method was in good agreement with soil sampling method.

Minirhizotron, to some extent, provided a method of observing roots over a long period of time [24, 25]. However, many studies have shown that application of minirhizotron disturbed both the roots and the soil [26, 27]. Joslin [28] showed that installation of minirhizotron resulted in excessive root proliferation, particularly near the soil surface, and it would take about 2 years for the trees to return to a new equilibrium. Itoh [29] proved that root growth path was changed with the installation of minirhizotron tube, and the correlation between root length density and real value became small with the increase of tube diameter. In addition, the installation of minirhizotron may cause problems such as affecting the compactness of soil and introducing light; the material of minirhizotron may disturb roots and soil; traditional minirhizotron also had difficulty in accurate observation of fine roots [18, 30, 31]. Therefore, the improved minirhizotron technology emerged as required.

Amato [32] developed a high quality low-cost digital microscope minirhizotron system with an amplifier and provided detailed building process. Cai [33] used acrylic glass, which had higher hardness, higher transparency, and less effect on root growth, proposed a method of installing minirhizotron tubes without digging soil so that root development could be monitored in naturally structured soils. Traditional research assumed that it was visible for cameras in the minirhizotron within 2–3 mm of soil [8, 22, 34]. But Taylor et al. believed that this assumed value was so large that the measured root diameter may well be smaller than the true value. His empirical algorithm assumed that the visible thickness is only 0.78 mm, and his method improved the accuracy of root diameter and biomass with captured images [26]. These methods mainly focused on the installation, image processing methods, and size and materials of minirhizotron. However, it is rarely reported that biological characteristics and spatial distribution characteristics of root system taken into account along with adaption of minirhizotron, to monitor roots effectively and reasonably.

In this paper, we explore how to improve the use of minirhizotron from two aspects: reducing the size of minirhizotron and improving installation validity and rationality with biological characteristics and spatial distribution characteristics of root system (*Capsicum annuum*). A kind of adaptive minirhizotron (microrhizotron) and processing systems, 1.5 cm^3^ in volume, was designed to observe roots dynamically. This microrhizotron system was able to focus on local and micro root changes in situ fast and accurate, and it can be used to observe roots in multiple points, which will provide more detail changes of roots in different points reliably for plant nutrition, plant physiology and ecology.

## Material and method

### Plant materials and growth conditions

*Capsicum annuum* (Bell Pell, Shouhe Co., Ltd, Shandong, China) seeds were sown in plug tray containing cultivated soil (organic matter: 478 g/1000 g, N + P + k: 6.75 mg/1000 g, probiotics: 90 µg/1000 g, trace element: 1.7 µg/1000 g, humic acid: 130 µg/1000 g, pH 6.5–6.8). After germinating in greenhouse in Nanjing Agricultural University, Engineering College (32°18′N, 118°46′E) for 18 days, pepper seedlings with two true leaves were transplanted to 3 experimental pots (1.2 m × 1.2 m × 0.4 m). The plant spacing was 25 cm and each plant was irrigated uniformly of 200 ml water every day with sprinkle from the field planting day. The soil (brown clay soil, organic matter: 318 g/1000 g, total N: 1.6 g/1000 g) in the pots was taken from local vegetable growing area, and was filtered 3 times with 1 cm opening to filter soil blocks larger than 1 cm^3^. The soil compaction was 0.81 kg/cm^2^ at the field planting day.

In the first experiment (root architecture 3D reconstruction), plants were sampled in 7 days, 13 days, 19 days, 25 days, 31 days and 39 days after field planting, 3 replicates. In the second experiment (interception probability in different installation patterns), there were totally 8 installation patterns, 12 replicates. In the third experiment (comparing the microrhizotron with soil sampling), 16 samples were used to compare microrhizotron obtained root indicators with manual measured root indicators.

### Adaptive minirhizotron

Since traditional minirhizotron was always large in diameter, it was not suitable to detect roots of shallow plant like peppers, which might cause great effects on root development. In order to observe roots development of shallow root plant in real time and multiple points, a kind of micro acquisition and processing system (mcrorhizotron) for multiple roots observation was proposed and designed in this paper.

**Fig. 1:**
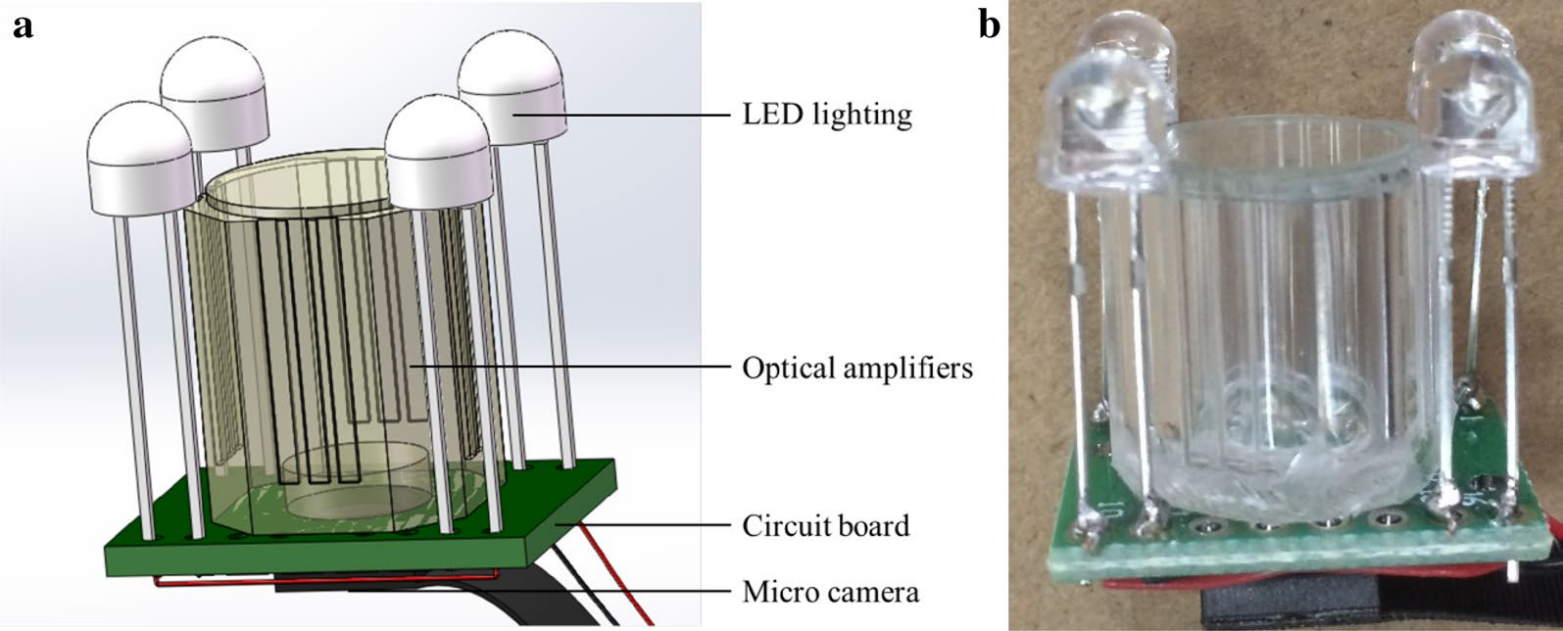
Structure diagram and photo of micro root observation system. **a** Structure diagram of microrhizotron. **b** Picture of microrhizotron

The mcrorhizotron consisted of a micro camera (HDMINICAM, Guangdong, China), an optical amplifier (amplified by 20 times), LED, a power supply and wireless module integrated in the camera, and image processing part (Fig. 1). The camera is 12 million pixel CMOS (complementary metal oxide semiconductor), resolution 1080p, angle of view 120°, working temperature − 20 °C − 80 °C, relative humidity 15–85%. Four LED light sources were installed on the top of the microrhizotron to illuminate imaging area. In order to prevent short circuit troubles, the circuit part of the system was sealed with hot melt glue and sprayed with waterproof paint for circuit board (A-2577, China) [35]. The size of the system was 1.1 cm × 1.1 cm × 1.2 cm. The collected root image was transmitted to the terminal receiving device (mobile phone or personal computer) through the WIFI signal formed by the wireless control module (Model: MEDIATEK MT7601).

### Image processing

**Fig. 2:**
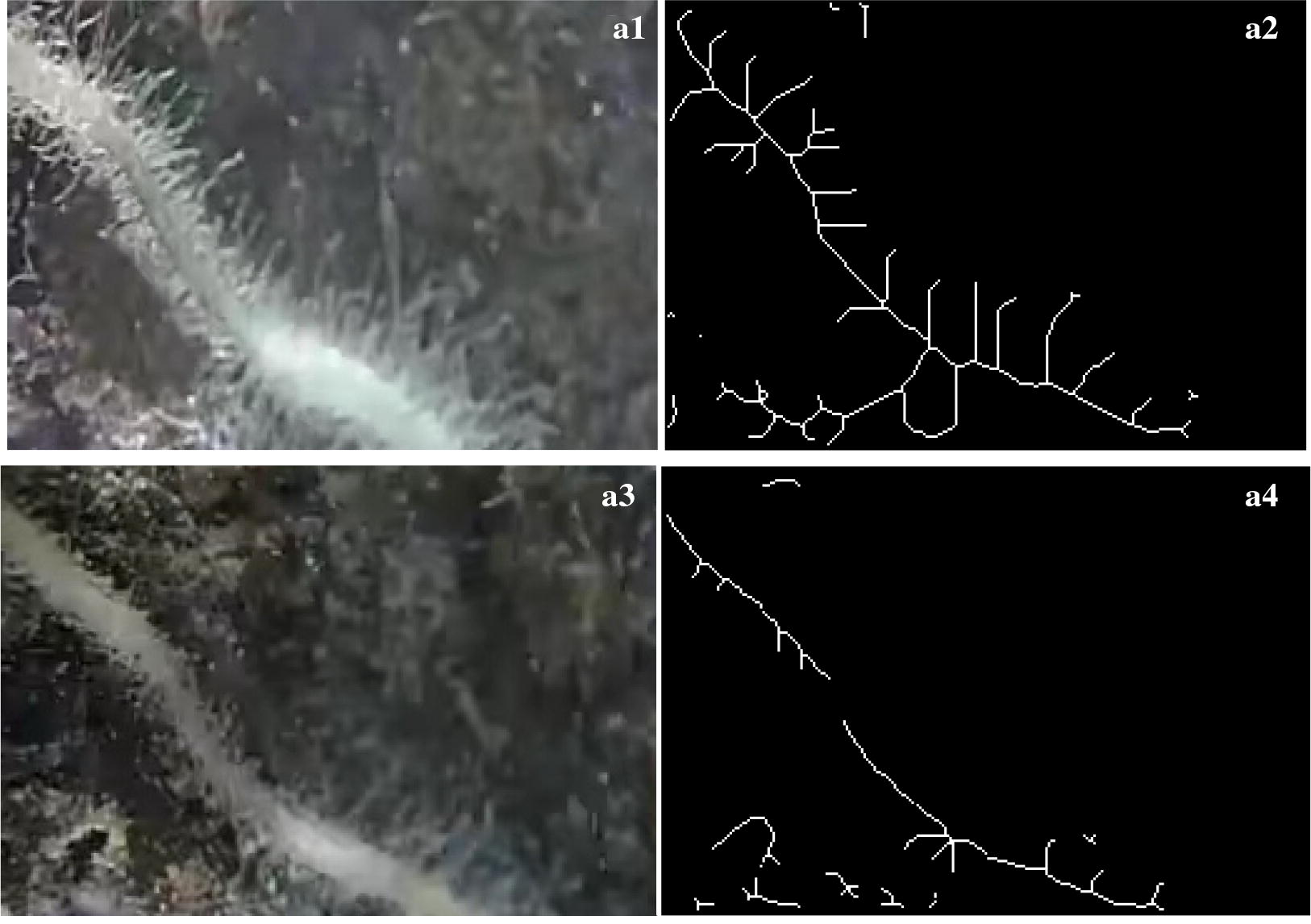
Source picture and processed image of roots. **a1** Source picture of roots observed in 28 days. **a2** Skeleton of a1. **a3** Source picture of roots observed in 60 days. **a4** Skeleton of a3

The JPEG files that contained the images of roots were used to determine the root length (including root hair and very fine roots) and root diameter. The root image was preprocessed through image normalization, enhancement, and median filter, and then through image morphology operation, skeleton image in single pixel were extracted [36, 37] as is shown in Fig. 2. In Fig. 2a1, a3 were the same root with different root hair or fine roots observed in different days. Figure 2a2, a4 were extracted root skeleton in single pixel. It was obvious that Fig. 2a2 had more and longer root hair or fine roots than that of Fig. 2a4. Root length was measured through calculating connections between adjacent pixels including diagonal connection or straight-line connection in skeleton image [37]. If two adjacent pixels is straight-line connection, the distance between the two pixels is 1; if it is diagonal connection, the distance is $\sqrt 2$. Code and script were developed by authors. However, in this way of automatic calculation, both the root axes and very fine root (root hairs) were included. And in root images (Fig. 2), fine root (root hairs) composed the most part. In order to analyze different root traits and to improve measuring accuracy, root axes and root hairs should be separated. Therefore, after the total root length was calculated automatically in skeleton image, root axes length was measured using ruler tools in Adobe Photoshop CS6 Extended. Root surface area was measured by counting the root pixels and calculating total area by multiplying area of each pixel based on the segmented binary image [35]. Root length and diameter was calculated by:

1 $$l = (\sqrt 2 N_{1} + N_{2} )K/{\text{f}}$$

2 $$S_{r} = K \times P/f$$

3 $$d = S_{r} /l$$

where l is root length, $S_{r}$ is root surface area, f is the magnification of the optical element, P is the total number of root pixels, K is the corresponding relation between the pixel distance on the image and the actual pixel distance, d is mean root diameter. N1 represents the number of pixels connected diagonally, and N2 represents the number of pixels connected horizontally and vertically (Additional files 1, 2).

### Root architecture 3D reconstruction and spatial distribution analysis

**Fig. 3:**
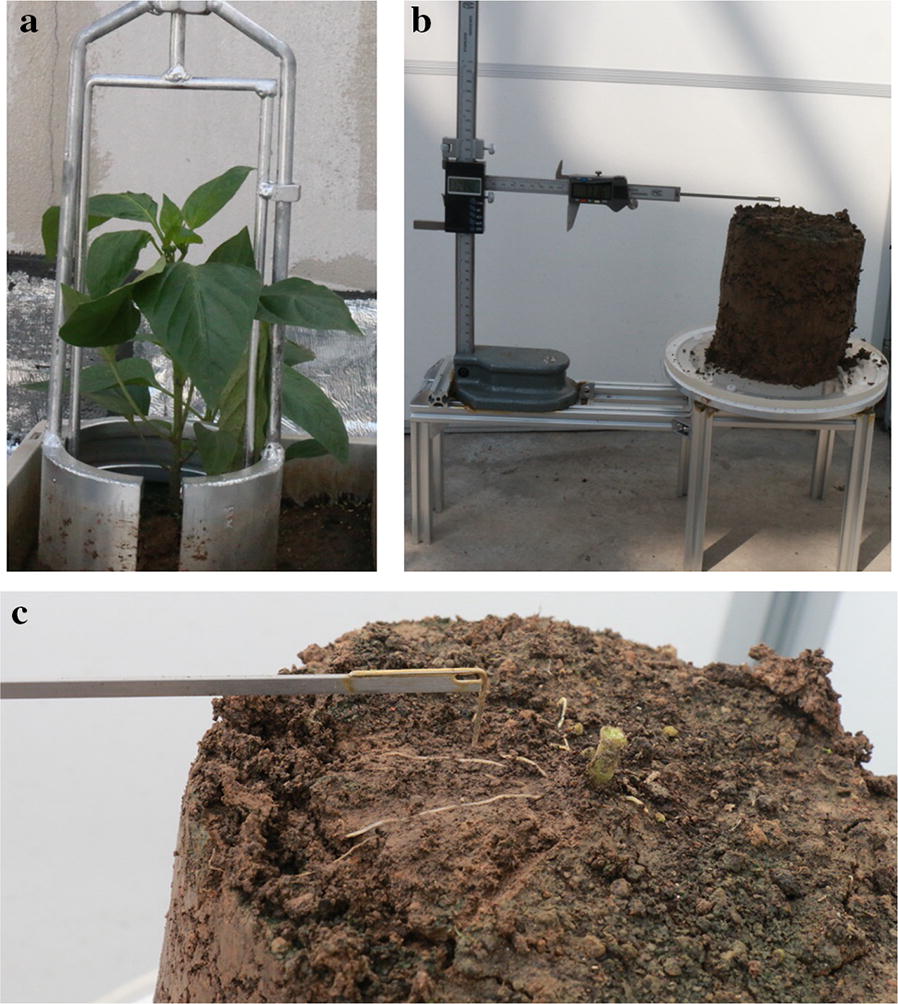
Root spatial structure measuring instrument. **a** Sampling of pepper roots. **b** Measuring instrument. **c** Measurement of lateral roots architecture

The root samples were excavated using a columnar sampler every 7 days (10 days after field planting, taking the center of the plant root system as the center, 10 cm as the radius (Fig. 3a)). The root architecture was measured using a three-dimensional caliper as shown in Fig. 3b. This instrument was mainly composed of height ruler, horizontal direction ruler, rotating disk ruler and laser centering device. First, the sample of the soil column and rotating disk were centered with a line laser, and then the top soil was gently removed with a small brush from the upper layer. The underlying soil of the root was preserved to make the root attached to it, so as not to destroy the original spatial position. The exposed root was measured with the tip of the three-dimensional caliper at each 2–3 mm interval, and it was cut off from the base after the last point was measured (Fig. 3c). The measured data were transformed from cylindrical coordinate system to Cartesian coordinate system, and the root system was reconstructed and analyzed with Matrix laboratory (version: 7.11.0.584). The code or script was developed by author.

**Fig. 4:**
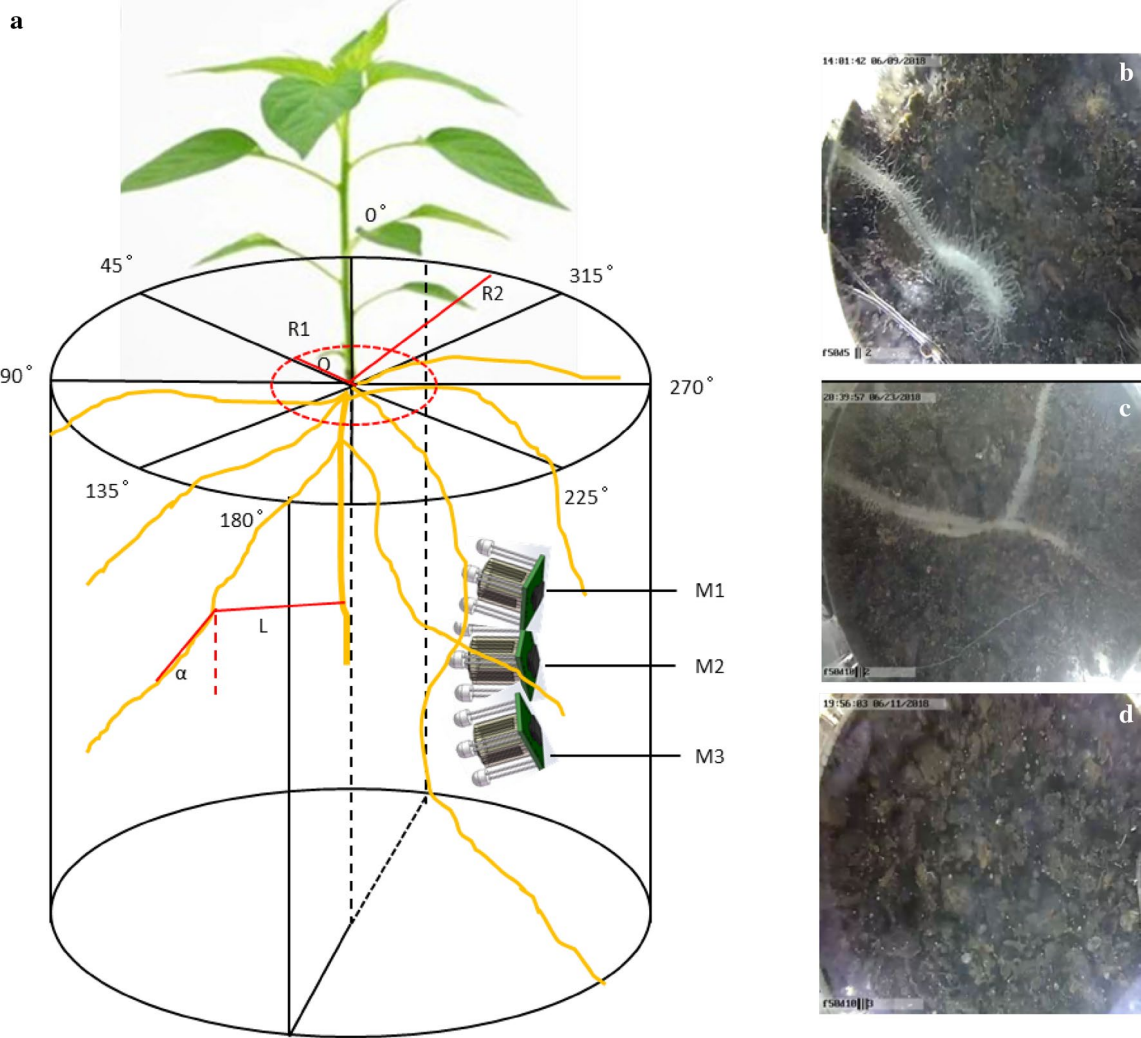
Installation patterns of microrhizotrons. **a** Installation patterns of microrhizotrons. M1, M2, M3 are microrhizotrons in different vertical angles and depth. α is vertical angle, L is lateral roots extension, and R1 and R2 are root length density sampling radius. **b**, **c**, and **d** Roots images from M1, M2 and M3

Roots of capsicum belong to diarch, and the lateral roots initiate from two opposite poles of protoxylem pole pericycle cell. Lateral roots are generally spaced along the longitudinal axis of the main root. Although it has long been known that lateral roots are symmetrically located, the spatial distribution of lateral roots after elongation remains unknown. In order to figure out root system architecture traits, the spatial structure after root elongation was further analyzed in circumferential direction, radial direction, and vertical direction (Fig. 4). α was used to characterize the angular change in the vertical direction, and it was the angle between lateral root (each elongation of 20 mm) and plant center line. L was used to characterize the circumferential spreading length of lateral roots, and it was the distance between lateral root (each elongation of 30 mm) and plant center line in horizontal direction.

Root length density was calculated every R = 10 mm radically by the formula as follows:

4 $${\text{RLD}} = \frac{{\mathop \sum \nolimits_{i = 1}^{n} l_{i} }}{V}$$

where RLD is root length density, $l_{i}$ is length of root i, and V is volume of the soil with roots.

### Installation validity and rationality of microrhizotron

In order to reduce the disturbance caused by installation, the microrhizotrons were presetted at the field planting day. 1.5 cm^3^ (1.1 × 1.1 × 1.2 cm) of soil was removed (replaced by microrhizotron), and the camera of the micro observation system was set carefully towards the roots. The rest of the soil was covered and compacted gently by rubber hammer to ensure that the soil compactness was consistent with the original growing soil environment. We declared a root interception success when the captured root length (root diameter) increased or decreased between t day and t + 1 day.

As is shown in Fig. 4, there were a lot of possibilities to set microrhizotrons. Circumferential position around the plant, installation radiuses of microrhizotron to the root center line, vertical angle and depth may all contribute to the effectiveness of microrhizotrons. For example, M1, M2 and M3 were set at random into soil. M1 was able to intercept with roots, and M2 would intercept with more; but M3 was further from roots, so this set of microrhizotron might be invalid in observing roots. Therefore, to improve the capture of roots, microrhizotrons should be presetted reasonably. And our solution was to install microrhizotrons based on biological characteristics and spatial distribution characteristics of root system, including determining optimal circumferential position, radical position and vertical angle.

### Comparing the microrhizotron with soil sampling

**Fig. 5:**
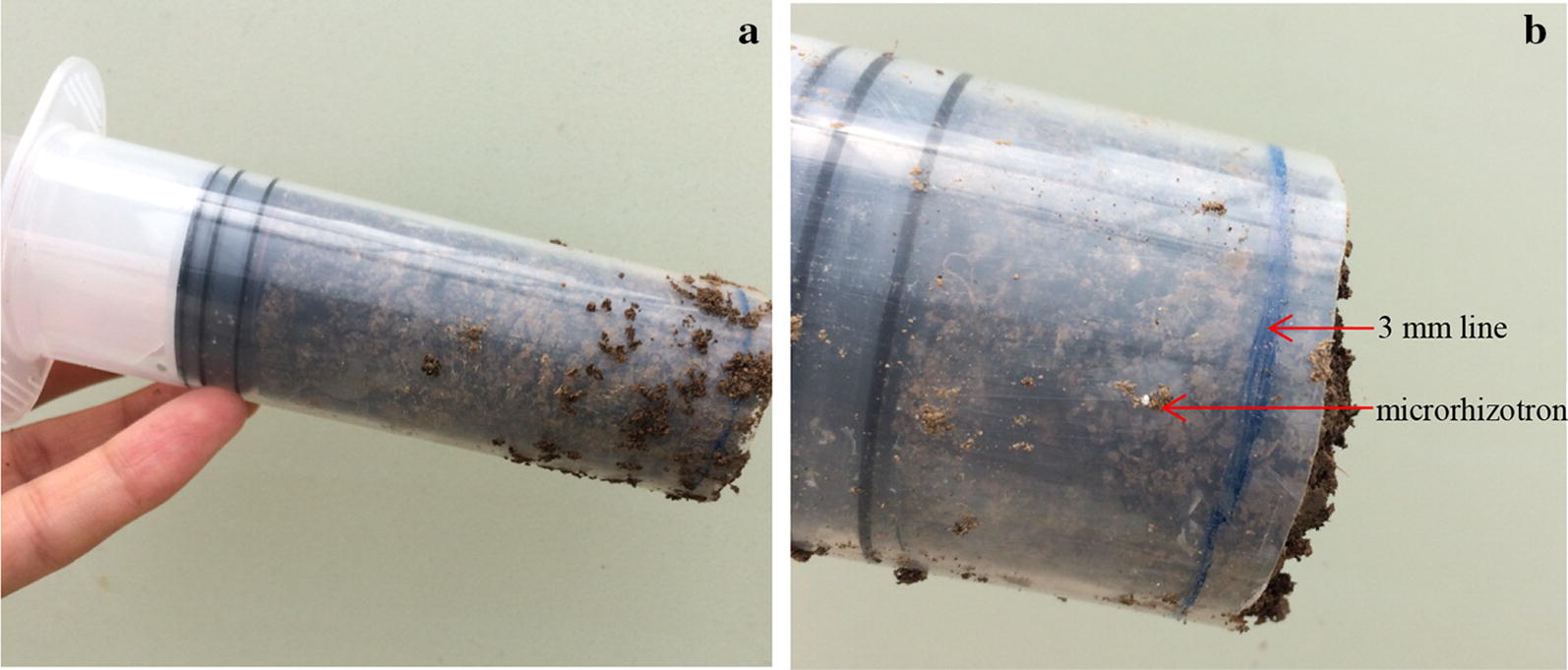
Soil sampling of roots opposite to the camera in 3 mm

Comparison experiment was conducted with soil sampling using the microrhizotron developed. In the experiment, each plant was installed with 2 microrhizotrons (opposite) in installation pattern horizontal angle 45° + radius 30 mm + vertical angle 45° (depth 50 mm) with 16 replicates. At four dates, root image was captured and processed by microrhizotron system. At the same time, soil samples were extracted with a cut syringe (diameter 2 cm) as is shown in Fig. 5. The syringe was used to extract soil cylinder opposite the camera. After the soil cylinder together with the microrhizotron was extracted, they were carefully put out and cut off layer by layer until 3 mm to the camera [24, 25], thus the roots in the view of camera was acquired. The roots were then washed carefully with distilled water and wiped with absorbent paper, and then measured root length and diameter manually with microcallipe.

## Results

### Three-dimensional reconstruction of capsicum root system

**Fig. 6:**
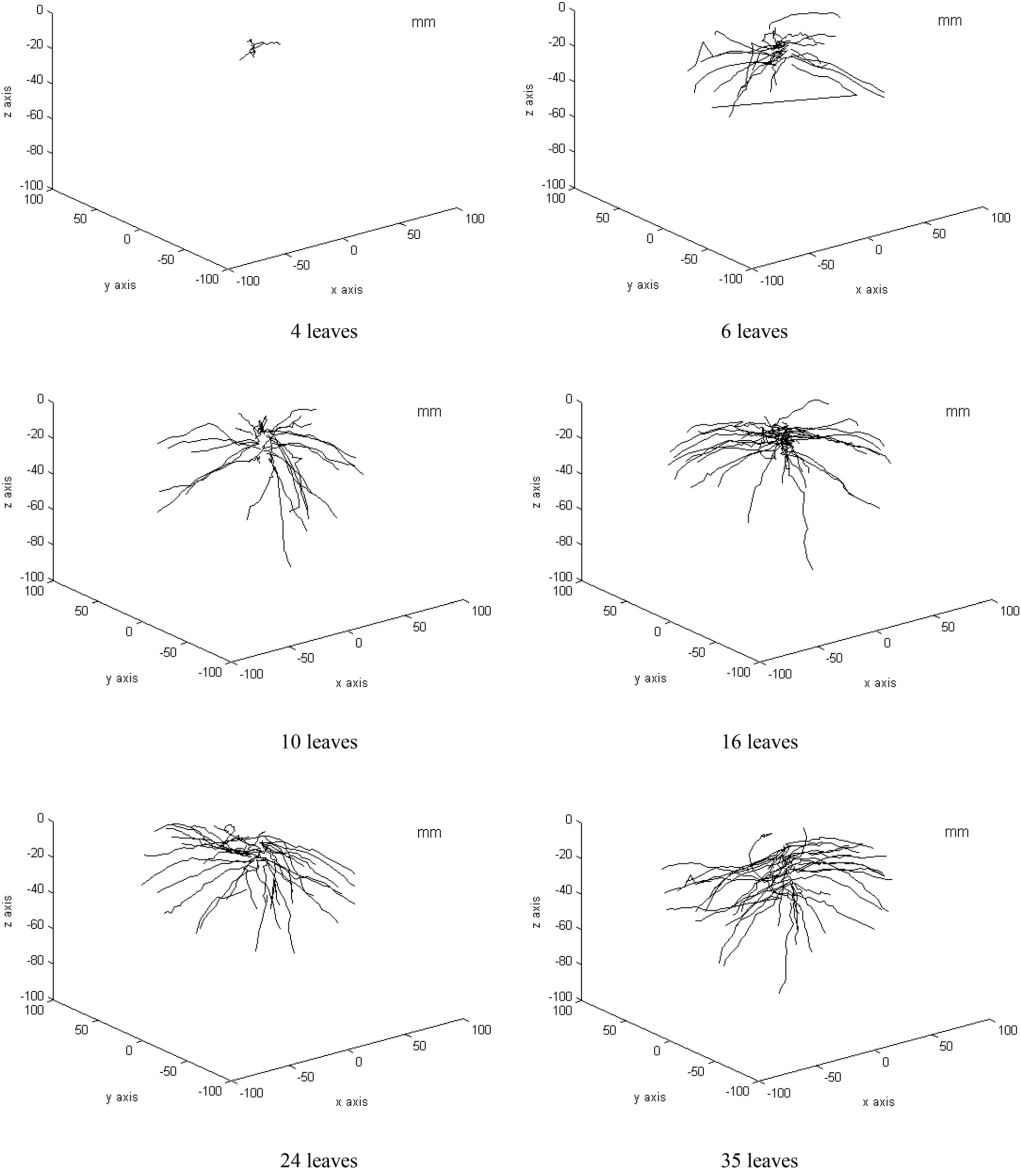
Root system architecture 3D reconstruction. 4, 6, 10, 16, 24 and 35, are numbers of true leaves, and they were sampled in 7 days, 13 days, 19 days, 25 days, 31 days and 39 days after field planting

Root 3D reconstruction for 4, 6, 10, 16, 24, and 35 leaves stage is shown in Fig. 6. Root system architecture depicted spatial configure of lateral roots, and root system in different stage showed very different architecture. There were only a small number (8 roots) of lateral roots at the 4 leaves stage, and these roots were very small and just existed within 5 cm soil layer. Roots in 6 leaves were much denser than that in 4 leaves. As the plant grew to 10 leaves, root number increased to 37; most roots spread in the top soil, and a few extended to deep soil. At the 35 leaves stage, there was only a slight increase in root number, but a large number of roots extended to soil layer deeper than 5 cm.

### Circumferential distribution of lateral roots after elongation

**Fig. 7:**
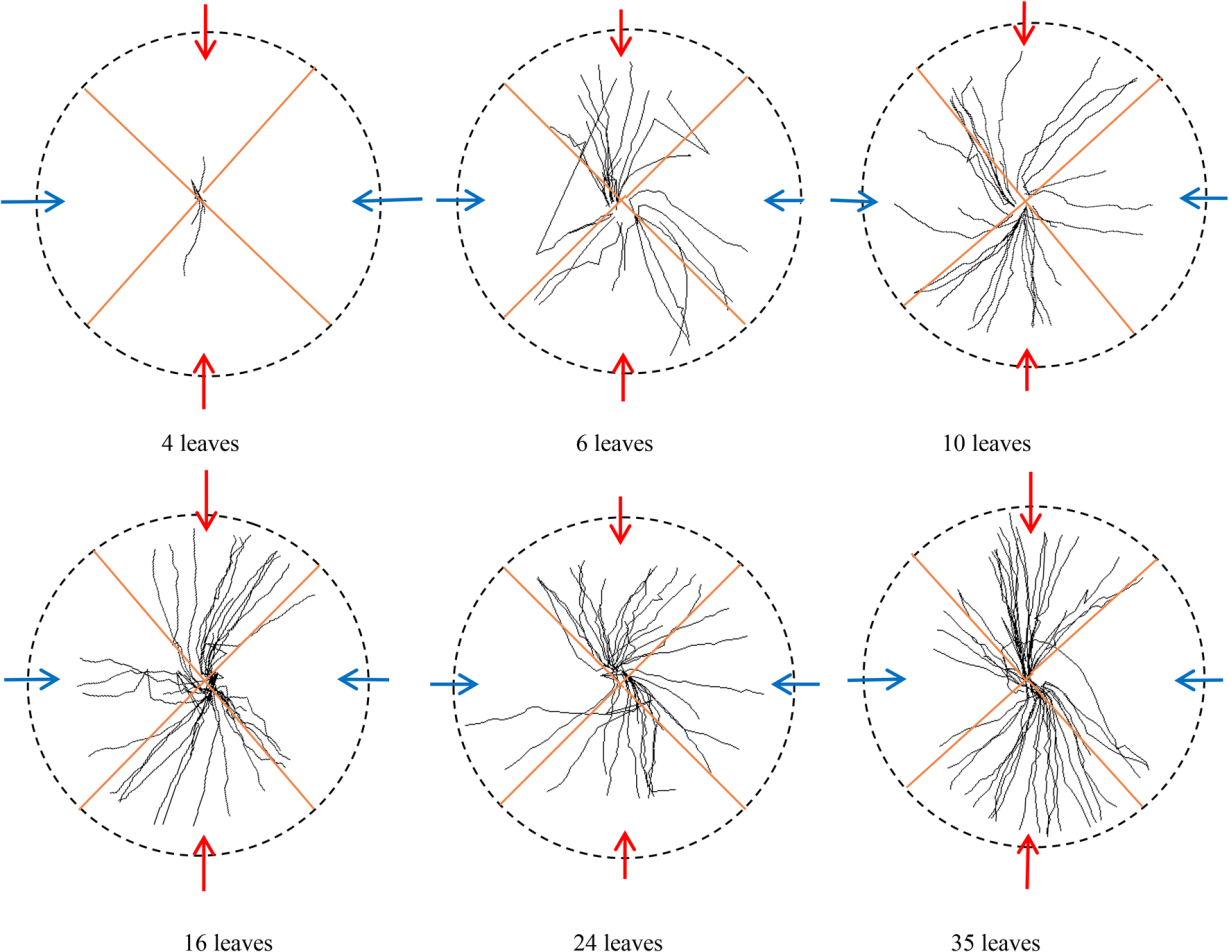
Lateral root gathering characteristics in vertical view

**Fig. 8:**
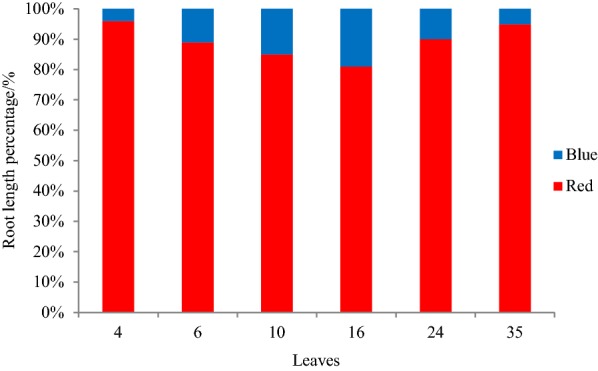
Lateral root gathering rate in vertical direction. Red represented root length density in red arrow area and blue represented root length density in blue arrow area

From vertical view, lateral roots distributed radiative along primary root. Two perpendicular lines were introduced to divide the roots into 4 parts. Each line was about 45° to initials of lateral roots. Interestingly, roots tended to gather in red arrow area, but in blue arrow area, roots were much less (Fig. 7). For understanding of spatial distribution of lateral roots after elongation, the circumferential gathering traits was quantified by calculating root length in red arrow area and in blue arrow area in Fig. 8. The result showed that lateral roots were highly focused in the red arrow area. The highest root length percentage of red arrow area in the 4 leaves stage reached 96%. Although in 16 leaves stage, the percentage was a little lower (84%) than the beginning, the average percentage was 90%, indicating highly gathering of lateral roots.

Pepper root system is diarch, and the lateral roots initiate from a patch of protoxylem pole pericycle cell, and are symmetrically located in two rows [38, 39]. To some extent, our findings implied that spatial distribution of lateral roots after elongation was also restricted to some range, and restricted horizontal angle was a highly-conserved mechanism shared by most lateral roots.

### Vertical angle and root extension

**Fig. 9:**
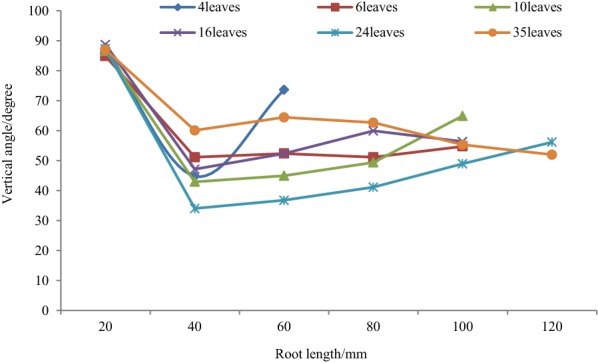
Vertical angle of lateral roots

**Fig. 10:**
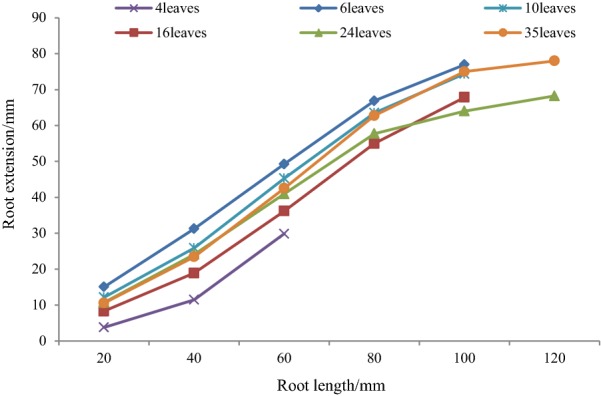
Extension of lateral roots

Vertical angle represented geotropism and deepen ability of lateral roots. As is depicted in Figs. 9 and 10, root length increased as plant leaves increased. The shortest root length was 60 mm in 4 leaves and extended to 120 mm in 35 leaves. For most lateral roots, vertical angles were between 80° and 90° at the initial position, almost perpendicular to the root center line. This indicated that lateral roots extended circumferentially at first to support the plant. Vertical angle since 20 mm was much lower than that of the beginning, and plant roots in different leaves showed different variation trend. Vertical angle at 4 leaves stage and 6 leaves stage seemed to be larger than that of 10 leaves stage, 16 leaves stage, and 24 leaves stage, but not that in 35 leaves stage. These differences might be caused by plant root development, since a smaller vertical implied lateral roots plunged into deep soil, which helped to search for water and nutrition, and to improve lodging resistance. Also, root phenotype was not only controlled by gene, but also by environment. Therefore, roots architecture may adapt appropriately to the environment. Although there was some difference as root exploring in soil, vertical angle of lateral roots was between 30° and 60° after elongation until 120 mm.

Root extension was the distance of root to plant center line. Individually, root extension had some difference, for example, root extension in 4 leaves was the lowest, and that in 6 leaves was the highest. This is because plant had individual difference, and also different reflection to environments. Generally, lateral roots extension linearly increased when root length was less than 80 mm. Then the increase slowed down, and reached peak value in root end. The longer the roots extent, the more water and nutrition it could access, and also more stable the plant would be. Therefore, the fast root extension in early days enabled a strong root basis for later development.

### Root length density distribution

Root length density is heterogeneous in soil in different depths and radius. As is shown in Table 1, the root length density near the root center was the largest. With the increase of sampling radius, root length density decreased in general. The root system was concentrated in the shallow soil within 5 cm in 4 leaves, 6 leaves, and 10 leaves stages. When plants had more than 16 leaves, root system gradually appeared in the soil layer of 10 cm, but compared with 5 cm soil layer, root length density was much smaller in 10 cm soil layer. Few roots were observed in depth more than 10 cm.

**Table 1:** Root length density distribution

Number of leaves	Depth (mm)	R (mm)								
		10	20	30	40	50	60	70	80	Average
4	0–50	0.637	0.056	0.015	0.010	0	0	0	0	0.090
	50–100	0	0	0	0	0	0	0	0	0
6	0–50	2.023	1.639	0.623	0.462	0.170	0.081	0	0.058	0.632
	50–100	0	0	0	0	0	0	0	0	0
10	0–50	2.978	1.070	0.433	0.261	0.113	0.064	0.037	0.017	0.622
	50–100	0	0	0.011	0.001	0	0	0	0	0.002
16	0–50	5.376	1.082	0.534	0.307	0.165	0.129	0.083	0.032	0.963
	50–100	0.065	0.039	0.019	0	0	0	0.009	0.010	0.018
24	0–50	5.599	2.067	0.618	0.224	0.082	0.041	0.019	0.000	1.081
	50–100	0	0.097	0	0	0	0	0	0.007	0.013
35	0–50	3.694	1.477	0.679	0.540	0.318	0.205	0.141	0.0516	0.888
	50–100	0	0	0.026	0.011	0	0	0	0	0.005

R referred to sampling radius

Average root length density was only 0.09 in 4 leaves, and it increased to 1.081 in 24 leaves stage. Compared with average value, root length density in R = 10 mm was much greater, and root length density in R > 40 mm was much smaller. Root length density between R = 20 mm and R = 40 mm was approximate to the average value. Interestingly, from time series root length density increased to the maximal value at 24 leaves, and then decreased. On one hand, this may be caused by circumferential expansion. As is shown in 35 leaves, although root length density near to the center decreased, it increased in large radius. On another hand, this may be related to root senescence and death, and the growth of the shoot, but still needs to be proved rigorously.

### Interception probability by different installation patterns according to root system architecture

According to the analysis of root initiate principle and root elongation discussed before, 8 installation patterns (12 replicates) for microrhizotron were tested and verified to determine high probability of root interception. They were the permutation and combination of 2 horizontal angles (45° and 90°), 2 radiuses of microrhizotron to the root center line (30 mm and 60 mm), and 2 vertical angles [18] (30° and 45°). Each plant was equipped with 3 microrhizotrons, and the depth of all the presets was 50 mm. It was convenient to observe root interception with microrhizotron every day, and in Fig. 9 we illustrated interception probability every 6 days.

**Fig. 11:**
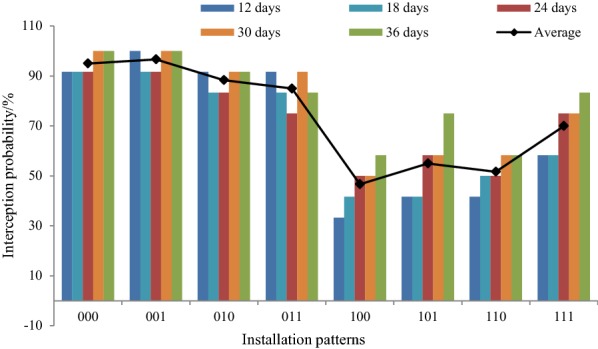
Probabilities of root interception under different installation patterns. *Notes* The first number in the combined number represents horizontal angle of microrhizotron to referred plane, where 0 is 45° and 1 is 90°. The second number in the combined number represents radius of microrhizotron to the root center line, where 0 is 30 mm and 1 is 60 mm. The last number in the combined number represents vertical angle of microrhizotron to the root center line, where 0 is 30° and 1 is 45°

As is shown in Fig. 11, probabilities of root interception under different installation patterns were greatly different. The highest interception probability appears in combination 001 with averaged value 96.7%, followed by 000 with probability 95%, and the lowest probability was only 46.7% in combination 100. Combination 0×× showed much higher interception probabilities than combination 1××, indicating that a smaller horizontal angle will make the camera intercept with more roots. Interception probabilities in combination ××1 was slightly higher than that of ××0, implying installation of 45° in vertical direction may be more proper for root interception in depth 50 mm.

From time sequence, averaged interception probabilities increased slightly from 68.7 to 81.2% as days increased. The reason for this was that the development and elongation of roots improved interception with microrhizotron.

It was also obvious in the figure that if installation of microrhizotron were at random, interception probability might be only 73.5%, while presetting according to root architecture features may increase this value to 96.7%. The results proved that interception probability could be improved through proper and right preset of microrhizotrons according to roots architecture features.

### Comparing root traits acquired from microrhizotron with soil sampling

As is shown in Tables 2 and 3, when root hair and very fine root were excluded, the relative error was 12.1% between microrhizotron and soil sampling in root length, and 15.4% in diameter. If root hair and very fine root was included, the relative increased high to 93.5% in root length and 48.9% in diameter. This was because most root hair and very fine root were broken and lost during soil sampling. So, in most cases, very fine roots could not be evaluated by soil sampling. However, excluding root hair and very fine root, average error of root length was 2.42 mm. Average root diameter error was 0.05 mm, and it was able to observe fine roots about 0.1 mm in diameter.

**Table 2:** Comparison of root length acquired from microrhizotron and soil sampling (mm)

	Ck	Tr1	Tr2	Er1	Er2		Ck	Tr1	Tr2	Er1	Er2
1	7.95	16.67	6.06	8.72	1.89	9	23.10	40.11	18.81	17.01	4.29
2	11.00	28.75	9.83	17.75	1.17	10	25.10	39.90	20.60	14.80	4.50
3	8.50	14.67	7.50	6.17	1.00	11	30.50	56.70	26.43	26.20	4.07
4	8.60	20.88	7.23	12.28	1.37	12	33.50	61.80	33.40	28.30	0.10
5	11.70	29.90	10.00	18.20	1.70	13	39.50	50.40	34.23	10.90	5.27
6	13.80	30.66	12.80	16.86	1.00	14	18.00	33.98	17.60	15.98	0.40
7	15.75	28.75	16.20	13.00	0.45	15	24.20	40.70	25.50	16.50	1.30
8	19.45	32.67	15.65	13.22	3.80	16	40.95	79.40	34.54	38.45	6.41

Tr1 represents root length including root hair acquired from microrhizotron, Tr2 represents root length excluding root hair acquired from microrhizotron. Ck represents root length acquired from soil sampling. Er1 is error between Tr1 and Ck, and Er2 is error between Tr2 and Ck

**Table 3:** Comparison of average diameter acquired from microrhizotron and soil sampling mm

	Ck	Tr1	Tr2	Er1	Er2		Ck	Tr1	Tr2	Er1	Er2
1	0.14	0.06	0.1	0.08	0.04	9	0.54	0.36	0.6	0.18	0.06
2	0.09	0.05	0.12	0.04	0.03	10	0.62	0.28	0.57	0.34	0.05
3	0.11	0.05	0.09	0.06	0.02	11	0.76	0.39	0.71	0.37	0.05
4	0.15	0.07	0.1	0.08	0.05	12	0.78	0.44	0.79	0.34	0.01
5	0.34	0.11	0.43	0.23	0.09	13	0.88	0.43	0.88	0.45	0
6	0.40	0.19	0.3	0.21	0.1	14	0.77	0.38	0.8	0.39	0.03
7	0.44	0.23	0.4	0.21	0.04	15	0.68	0.33	0.67	0.35	0.01
8	0.33	0.21	0.42	0.12	0.09	16	0.45	0.29	0.39	0.16	0.06

Tr1 represents root length including root hair acquired from microrhizotron, Tr2 represents root length excluding root hair acquired from microrhizotron. Ck represents root length acquired from soil sampling. Er1 is error between Tr1 and Ck, and Er2 is error between Tr2 and Ck

## Discussion

The aim of this work was to adapt minirhizotron to a microrhizotron system that enables for multipoint observation for local and micro root changes, and investigated 3D distribution traits of *Capsicum annuum* under regular fertilizer and water strategy to ensure installation validity and rationality. Performances with different installation patterns of the microrhizotron system were validated, and root length and diameter were compared with soil samplings.

The microrhizotron system is a good compromise between the nondestructive detection and handling capacities of dynamic observation and the advantage of multipoint observation for roots. A big advantage of the microrhizotron is that they facilitate studying the response of roots in different positions, rather than watching in long tube and large diameter from top to bottom (from bottom to top), and obtaining roots image limited to the tube wall. For example, root length density in different depths and radius to root center line could be accurately acquired with the designed microrhizotron. Besides, it could also be applied to study on other root system traits (root hairs), and the plasticity and the dynamic alteration of root growth to environment and irrigation strategy.

In the root architecture reconstruction, it was obvious that capsicum root existed in shallow soil less than 10 cm. Our result was not in consistence with Kong Q, in which root depth of pepper reached 40 cm [20]. This was attributed to different soil compactness in Kong Q’s study (1.4 g/cm^3^) and in our study (1.55 g/cm^3^), since larger soil compactness made root harder to develop in depth [3, 40]. Disregarding the genetic variations, root development were affected by soil compactness and other soil environment (e.g. temperature, water holding capacity, hydraulic conductivity, porosity, pore size distribution and oxygen availability) [41, 42]. Therefore, for shallow root plants or roots constrained to shallow soil layer, microrhizotron would be more flexible, small and delicate for in situ observation.

Remarkably, from vertical view (Fig. 7), lateral roots are regularly initiating from two lines along primary. This agrees with the organization patterns of vascular bundles in dicot plants that vascular tissue exhibits diarch symmetry and only contains two xylem poles [38]. Quantification of root architecture in horizontal direction showed that root elongation was to some extent regulated to the two lines alone primary root. Although a small part slightly exceeded the angle, maybe caused by water and fertilizer inhomogeneity, or root competition, most lateral roots were constrained to an angle near to the initial position. In vertical direction, angles were larger at initial position and in shallow depth, and it became smaller when roots began to elongate. These results indicated that circumferential position and angles should be taken into account when presetting microrhizotron for dicot, *Capsicum annuum* especially in this research.

Installation has long been a problem in the use of minirhizotron [43]. Angle of the tube ranges from 0° to 90°, although the installation angles of 30° [44, 45] and 45° [46, 47] occur more frequently, there is no standard or scientific proof by now. In this study, by measuring the root architecture traits, we proved that in depth 50 mm, vertical angle 45° performed better than vertical angle 30° to intercept with roots. There were some difference between traditional minirhizotron and the adaptive minirhizotron described in this paper. For traditional minirhizotron, many studies have reported that installation of minirhizotron resulted in excessive root proliferation, particularly near the soil surface [26, 33, 48]. But in our study, no excessive root proliferation was observed. The new problem it faced was that microrhizotron sometimes failed to intercept with roots when preset at random. This may well be caused by the very small size of the microrhizotron, which reduce contact between roots and camera. Effective installation patterns to intercept with more roots were proved in this research. Our method was to quantify the spatial distribution of roots under conventional water and fertilizer treatments, and to install them based on that knowledge. 3D distribution traits of *Capsicum annuum* under regular fertilizer and water strategy could ensure installation validity and rationality effectively.

During plant root exploration in soil, plants may adapt their roots architecture to water, nutrients and environment. Therefore, observation dynamically over a long period involved in this process is crucial for improving plant growth under varying environmental stimuli. Our results clearly showed that microrhizotron is an ideal system for observation of roots compared with soil sampling when root hair and very fine root was excluded. Although root hair and very fine root cannot be evaluated with soil sampling method, it provides a multipoint, and nondestructive method to observe fine root and root hairs changes by day to day comparison. The limit of this study is that root observation with this kind of microrhizotron focused on local and subtle changes, and root system architecture traits were quantified with limited genotype and varieties. In the future study, observation of the root system architecture remains to combine microrhizotron with other methods, where the microrhizotron is used to focus on micro changes of roots, and method such as X-CT is used to acquire 3D root architecture.

## Conclusion

A new adaptive minirhizotron has been established for non-destructive and multipoint observation on roots, and its application and installation patterns has been suggested according to root architecture traits. The setup requires no sophisticated instruments, has a relatively small volume, and allowed in situ, rapid, non-destructive, multipoint analysis of root angle with minimal disturbance to plant growth. Root system architecture traits of *Capsicum annuum* was quantified by analyzing circumferential distribution, vertical angle, depth and root extension, which allowed for a more suitable and effective installation pattern of microrhizotrons. The preset validity could be highly improved by this method. The presented microrhizotron can be used to study a wide range of research questions on a small scale, for example, for quantitative trait locus analysis, root width changes, and root hair growth.

## Additional files

**Additional file 1.** Script for root system architecture construction and analysis.

**Additional file 2.** Script for root image processing.

## Abbreviations

X-CT: X-ray computed tomography
NMR: nuclear magnetic resonance
