# Supplementary material for: Adaptive minirhizotron for pepper roots observation and its installation based on root system architecture traits
Source: Plant Methods. 2019 Mar 23;15:29. doi: 10.1186/s13007-019-0414-z (PMC6431063; doi:10.1186/s13007-019-0414-z)
Supplement: Supplementary file 1 — Additional file 1. Script for root system architecture construction and analysis. [file 13007_2019_414_MOESM1_ESM.docx]

**% root image processing**

clear

clc

A=imread('E:\PHD\source image a1.jpg');

figure(1),imshow(A);

title('Source image'); % source picture

B=rgb2gray(A);

maxi=double(max(max(B))); %maximum gray

mini=double(min(min(B))); %minimum gray

range=maxi-mini; %gray range

BB=((double(B)-mini)./range)*255; % image normalization

BB=uint8(B);

C=imadjust(BB,[],[0 1]);

figure(2),imhist(C);

title('Source histogram');

D=imadjust(BB,[],[0.33 0.88]); % photographic enhancement by adjusted histogram

figure(3),imhist(D);

title('Adaptive histogram');

figure(4),imshow(D);

title('gray image');

E=im2bw(D,0.55); %Image binarization (the same installation point with the same threshold throughout the experimental period.)

F=medfilt2(E,[8 8]); % median filtering

figure(5),imshow(F);

title('Medfilte image');

I=bwmorph(F,'skel',Inf); % Image thinning

figure(6),imshow(I);

title('Skel image');

%pixel counting, pixel direction code and length calculating

[m,n]=size(I);

q=2^0.5;

sum=1;

for i=1:m

for j=1:n

%pixel in the first row

if (i==1)

if (I(i,j))

if (j==1) % %first row and first column

if (I(i,j+1))

sum=sum+1;

end

if (I(i+1,j))

sum=sum+1;

end

if (I(i+1,j+1))

sum=sum+q;

end

else

if (j==n) %first row and the last column

if (I(i+1,j-1))

sum=sum+q;

end

if (I(i+1,j))

sum=sum+1;

end

else % first row and other columns

if (I(i,j+1))

sum=sum+1;

end

if (I(i+1,j-1))

sum=sum+q;

end

if (I(i+1,j))

sum=sum+1;

end

if (I(i+1,j+1))

sum=sum+q;

end

end

end

end

else

% last column

if (i==m)

if (I(i,j))

if (j==n) %last row and last column

else

if (I(i,j+1))

sum+1;

end

end

end

else

% other rows

if (I(i,j))

if (j==1) %first column

if (I(i,j+1))

sum=sum+1;

end

if (I(i+1,j))

sum=sum+1;

end

if (I(i+1,j+1))

sum=sum+q;

end

else

if (j==n) %last column

if (I(i+1,j-1))

sum=sum+q;

end

if (I(i+1,j))

sum=sum+1;

end

else %ohter columns

if (I(i,j+1))

sum=sum+1;

end

if (I(i+1,j-1))

sum=sum+q;

end

if (I(i+1,j))

sum=sum+1;

end

if (I(i+1,j+1))

sum=sum+q;

end

end

end

end

end

end

end

end
