# Supplementary material for: Adaptive minirhizotron for pepper roots observation and its installation based on root system architecture traits
Source: Plant Methods. 2019 Mar 23;15:29. doi: 10.1186/s13007-019-0414-z (PMC6431063; doi:10.1186/s13007-019-0414-z)
Supplement: Supplementary file 2 — Additional file 2. Script for root image processing. [file 13007_2019_414_MOESM2_ESM.docx]

**% root construction**

clear

clc

in_path='E:\phD\rootreconstrcution\experiment1\';

out_path='E:\phD\rootresconstruction\experiment1\result\';

[Type Sheet Format]=xlsfinfo(' E:\phD\rootreconstrcution\experiment\data1.xlsx');

sheets=max(size(Sheet));

for i=1:sheets;

I=xlsread([in_path 'data1.xlsx'],i);

X=I(:,5); % X coordinate in spatial coordinates (transferred from Cylindrical coordinates )

Y=I(:,6); %Y coordinate in spatial coordinates (transferred from Cylindrical coordinates )

Z=I(:,7)-100; %Y coordinate in spatial coordinates (transferred from Cylindrical coordinates )

figure(1),plot3(X,Y,Z,'k');xlabel('x axis');ylabel('y axis');zlabel('z axis');

hold on;

I=[];X=[];Y=[];Z=[];

end

xlswrite([out_path ' reconstrcution.png']);

view(-37.5,30);print(gcf,'-dpng',' E:\phD\rootresconstruction\experiment1\result\3Dreconstrcution.png '); % 3D reconstrcution

axis([-100 100 -100 100 -100 0]);

view(180,90);print(gcf,'-dpng',' E:\phD\rootresconstruction\experiment1\result\vertical.png '); % vertical view(90,0);print(gcf,'-dpng',' E:\phD\rootresconstruction\experiment1\result\side_view.png '); % side view

view(180,0);print(gcf,'-dpng',' E:\phD\rootresconstruction\experiment1\result\front_view.png ');

**% vertical angle analysis**

clear

clc

in_path=' E:\phD\rootreconstrcution\experiment1\';

out_path=' E:\phD\rootreconstrcution\experiment1\result\';

[Type Sheet Format]=xlsfinfo(' E:\phD\rootreconstrcution\experiment1\data1.xlsx');

sheets=max(size(Sheet));

for j=1:sheets

I=xlsread([in_path ' data1.xlsx '],j);

I=double(I);

X1=I(:,1); %z

X2=I(:,2); %r

X3=I(:,3); %angle

X4=I(:,4); % radian

X5=I(:,5); %x

X6=I(:,6); %y

X7=I(:,7); %z

[r,c]=size(I);

X8=zeros(r,1); %distance to previous point (square value)

X9=zeros(r,1); % distance to previous point

X10=zeros(r,1); % Horizontal stretch (distance to centerline)

X11=zeros(r,1); % Vertical dashed line length

X12=zeros(r,1); % Length of oblique edges of an angular triangle

X13=zeros(r,1); %cosα¨

X14=zeros(r,1); %angle of α

for i=1:(r-1)

X8(i+1,1)=(X5(i+1,1)-X5(i,1)).^2+(X6(i+1,1)-X6(i,1)).^2+(X7(i+1,1)-X7(i,1)).^2;

X9(i+1,1)=sqrt(X8(i+1,1));

X10(i+1,1)=sqrt((X5(i+1,1).^2+(X6(i+1,1)).^2));

end

II=[X1 X2 X3 X4 X5 X6 X7 X8 X9 X10];

%II=uint8(II);

xlswrite([out_path ' vertical_analysis1.xlsx'],II,j);

%Selecting points at approximately 20mm intervals

k=1; temp=1;

sum=0;

X11(1,1)=abs(X6(1,1)); % length of the vertical dashed line

X12(1,1)=sqrt((X5(1,1)).^2+(X6(1,1)).^2+(X7(1,1)).^2); % length of the diagonal side of the right triangle

X13(1,1)=X11(1,1) ./ X12(1,1); %cosα

X14(1,1)=acosd(X13(1,1)); %angle of α

bait=[]; bait(1,1)=X14(1,1);

for i=2:r

sum=sum+X9(i,1);

if (sum>=20)

sum=0;

X11(i,1)=sqrt((X6(i,1)-X6(temp,1)).^2);

X12(i,1)=sqrt((X5(i,1)-X5(temp,1)).^2+(X6(i,1)-X6(temp,1)).^2+(X7(i,1)-X7(temp,1)).^2);

X13(i,1)=X11(i,1) ./ X12(i,1);

X14(i,1)=acosd(X13(i,1));

temp=i;

k=k+1;

bait(k,1)=X14(i,1);

end

end

xlswrite([out_path ' vertical_analysis2.xlsx '],bait,j);

end

**% root extension**

clear

clc

in_path=' E:\phD\rootreconstrcution\experiment1\';

out_path=' E:\phD\rootreconstrcution\experiment1\result\ ';

[Type Sheet Format]=xlsfinfo(' E:\phD\rootreconstrcution\experiment1\data1.xlsx');

sheets=max(size(Sheet));

for j=1:sheets

I=xlsread(in_path ' root_extension.xlsx ',j);

I=double(I);

X1=I(:,1); %z

X2=I(:,2); %r

X3=I(:,3); %angle

X4=I(:,4); %radian

X5=I(:,5); %x

X6=I(:,6); %y

X7=I(:,7); %z

[r,c]=size(I);

X8=zeros(r,1); % used to store the square distance to the last point

X9=zeros(r,1); % used to store the distance to the last point

X10=zeros(r,1); % used to store the horizontal distance to the core

X11=zeros(r,1); % opposite side of β

X12=zeros(r,1); %cosβ

X13=zeros(r,1); %βradian

X14=zeros(r,1); %βangle

for i=1:(r-1)

X8(i+1,1)=(X5(i+1,1)-X5(i,1)).^2+(X6(i+1,1)-X6(i,1)).^2+(X7(i+1,1)-X7(i,1)).^2;

X9(i+1,1)=sqrt(X8(i+1,1));

X10(i+1,1)=sqrt((X5(i+1,1).^2+(X6(i+1,1)).^2));

X11(i+1,1)=sqrt((X5(i+1,1)-75).^2+(X6(i+1,1)).^2);

X12(i+1,1)=(5625+(X10(i+1,1)).^2-(X11(i+1,1)).^2)/(2*75*(X10(i+1,1)));

X13(i+1,1)=acos(X12(i+1,1));

X14(i+1,1)=acosd(X12(i+1,1));

end

II=[X1 X2 X3 X4 X5 X6 X7 X8 X9 X10 X11 X12 X13 X14];

%II=uint8(II);

xlswrite([out_path ' root_extension1.xlsx '],II,j);

%% Take points at approximately 20mm intervals

k=1;

sum=0;

L=[]; L(1,1)=X10(2,1); % storage of extension laterally

bait=[]; bait(1,1)=X14(2,1); %βangle

for i=2:r

sum=sum+X9(i,1);

if (sum>=20)

sum=0;

k=k+1;

L(k,1)=X10(i,1);

bait(k,1)=X14(i,1);

end

end

xlswrite([out_path 'root_extension2.xlsx '],L,j);

end

**% root length in different radius**

clear

clc

in_path=' E:\phD\rootreconstrcution\experiment1\';

out_path=' E:\phD\rootreconstrcution\experiment1\result';

[Type Sheet Format]=xlsfinfo(' E:\phD\rootreconstrcution\experiment1\data1.xlsx');

sheets=max(size(Sheet));

L01=0; L01_1=0; L01_2=0; %the total root length from the core horizontal distance 0-10mm, _1 and _2 respectively represent vertical 5-10cm and 10-15cm.

L02=0; L02_1=0; L02_2=0; %the total root length 10-20mm to the center

L03=0; L03_1=0; L03_2=0; %the total root length 20-30mm to the center

L04=0; L04_1=0; L04_2=0; %the total root length 30-40mm to the center

L05=0; L05_1=0; L05_2=0; %the total root length 40-50mm to the center

L06=0; L06_1=0; L06_2=0; %the total root length 50-60mm to the center

L07=0; L07_1=0; L07_2=0; %the total root length 60-70mm to the center

L08=0; L08_1=0; L08_2=0; %the total root length 70-80mm to the center

initial=0;

I=xlsread([in_path 'data1.xlsx'],1);

initial=I(1,1); %origin

initial_x=I(1,5);

initial_y=I(1,6);

for j=1:sheets

I=xlsread([in_path 'data1.xlsx'],j);

I=double(I);

X1=I(:,1); %z

X2=I(:,2); %r

X3=I(:,3); %rad

X4=I(:,4); %radian

X5=I(:,5); %x

X6=I(:,6); %y

X7=I(:,7); %z

[r,c]=size(I);

X8=zeros(r,1); %used to store the distance to the previous point(square)

X9=zeros(r,1); %used to store the distance to the previous point

L=zeros(r,1); %used to store the horizontal distance to the center

for i=1:(r-1)

X8(i+1,1)=(X5(i+1,1)-X5(i,1)).^2+(X6(i+1,1)-X6(i,1)).^2+(X7(i+1,1)-X7(i,1)).^2;

X9(i+1,1)=sqrt(X8(i+1,1));

L(i+1,1)=sqrt((X5(i+1,1)-X5(1,1)).^2+(X6(i+1,1)-X6(1,1)).^2);

end

%It is adopted when the horizontal distance to the center is approximately 10mm.

sum=0;

for i=2:r

%depth 5cm

if ((abs(X1(i,1)-initial))<=50)

sum=sqrt((X5(i,1)-initial_x).^2+sqrt((X6(i,1)-initial_y).^2));

if (sum<=10)

L01=L01+X9(i,1);

end %Horizontal 0-1cm

if (sum>10 & sum<=20)

L02=L02+X9(i,1);

end %Horizontal 1-2cm

if (sum>20 & sum<=30)

L03=L03+X9(i,1);

end %Horizontal 2-3cm

if (sum>30 & sum<=40)

L04=L04+X9(i,1);

end %Horizontal 3-4cm

if (sum>40 & sum<=50)

L05=L05+X9(i,1);

end %Horizontal 4-5cm

if (sum>50 & sum<=60)

L06=L06+X9(i,1);

end %Horizontal 5-6cm

if (sum>60 & sum<=70)

L07=L07+X9(i,1);

end %Horizontal 6-7cm

if (sum>70 & sum<=80)

L08=L08+X9(i,1);

end %Horizontal 7-8cm

end

%depth 5-10cm

if ((abs(X1(i,1)-initial))>50 & (abs(X1(i,1)-initial))<=100)

sum=sqrt((X5(i,1)-initial_x).^2+sqrt((X6(i,1)-initial_y).^2));

if (sum<=10)

L01_1=L01_1+X9(i,1);

end %Horizontal 1cm vertical 5cm's root length

if (sum>10 & sum<=20)

L02_1=L02_1+X9(i,1);

end %Horizontal 2cm vertical 5cm's root length

if (sum>20 & sum<=30)

L03_1=L03_1+X9(i,1);

end %Horizontal 3cm vertical 5cm's root length

if (sum>30 & sum<=40)

L04_1=L04_1+X9(i,1);

end %Horizontal 4cm vertical 5cm's root length

if (sum>40 & sum<=50)

L05_1=L05_1+X9(i,1);

end %Horizontal 5cm vertical 5cm's root length

if (sum>50 & sum<=60)

L06_1=L06_1+X9(i,1);

end %Horizontal 6cm vertical 5cm's root length

if (sum>60 & sum<=70)

L07_1=L07_1+X9(i,1);

end %Horizontal 7cm vertical 5cm's root length

if (sum>70 & sum<=80)

L08_1=L08_1+X9(i,1);

end %Horizontal 8cm vertical 5cm's root length

end

%Vertical 10-15cm

if ((abs(X1(i,1)-initial))>100 & (abs(X1(i,1)-initial))<=150)

sum=sqrt((X5(i,1)-initial_x).^2+sqrt((X6(i,1)-initial_y).^2));

if (sum<=10)

L01_2=L01_2+X9(i,1);

end %Horizontal 0-1cm

if (sum>10 & sum<=20)

L02_2=L02_2+X9(i,1);

end %Horizontal 1-2cm

if (sum>20 & sum<=30)

L03_2=L03_2+X9(i,1);

end %Horizontal 2-3cm

if (sum>30 & sum<=40)

L04_2=L04_2+X9(i,1);

end %Horizontal 3-4cm

if (sum>40 & sum<=50)

L05_2=L05_2+X9(i,1);

end %Horizontal 4-5cm

if (sum>50 & sum<=60)

L06_2=L06_2+X9(i,1);

end %Horizontal 5-6cm

if (sum>60 & sum<=70)

L07_2=L07_2+X9(i,1);

end %Horizontal 6-7cm

if (sum>70 & sum<=80)

L08_2=L08_2+X9(i,1);

end %Horizontal 7-8cm

end

end

end

final_data=zeros(8,3);

final_data(:,1)=[L01;L02;L03;L04;L05;L06;L07;L08];

final_data(:,2)=[L01_1;L02_1;L03_1;L04_1;L05_1;L06_1;L07_1;L08_1];

final_data(:,3)=[L01_2;L02_2;L03_2;L04_2;L05_2;L06_2;L07_2;L08_2];

xlswrite([out_path 'root_length.xlsx'],final_data);

**% circumferential distribution analysis**

clear

clc

in_path=' E:\phD\rootreconstrcution\experiment1\';

out_path=' E:\phD\rootreconstrcution\experiment1\result\';

[Type Sheet Format]=xlsfinfo(' E:\phD\rootreconstrcution\experiment1\data1.xlsx');

sheets=max(size(Sheet));

for j=1:sheets

I=xlsread(in_path ' circumferential distribution analysis.xlsx ',j);

I=double(I);

X1=I(:,1); %z

X2=I(:,2); %r

X3=I(:,3); %rad

X4=I(:,4); %radian

X5=I(:,5); %x

X6=I(:,6); %y

X7=I(:,7); %z

[r,c]=size(I);

X8=zeros(r,1); % used to store the square distance to the last point

X9=zeros(r,1); % used to store the distance to the last point

X10=zeros(r,1); % used to store the horizontal distance to the core

X11=zeros(r,1); %opposite side of β

X12=zeros(r,1); %cosβ

X13=zeros(r,1); %βradian

X14=zeros(r,1); %βangle

for i=1:(r-1)

X8(i+1,1)=(X5(i+1,1)-X5(i,1)).^2+(X6(i+1,1)-X6(i,1)).^2+(X7(i+1,1)-X7(i,1)).^2;

X9(i+1,1)=sqrt(X8(i+1,1));

X10(i+1,1)=sqrt((X5(i+1,1).^2+(X6(i+1,1)).^2));

X11(i+1,1)=sqrt((X5(i+1,1)-75).^2+(X6(i+1,1)).^2);

X12(i+1,1)=(5625+(X10(i+1,1)).^2-(X11(i+1,1)).^2)/(2*75*(X10(i+1,1)));

X13(i+1,1)=acos(X12(i+1,1));

X14(i+1,1)=acosd(X12(i+1,1));

end

II=[X1 X2 X3 X4 X5 X6 X7 X8 X9 X10 X11 X12 X13 X14];

%II=uint8(II);

xlswrite([out_path ' circumferential analysis1.xlsx'],II,j);

%% Take points at approximately 20mm intervals

k=1;

sum=0;

L=[]; L(1,1)=X10(2,1); %Storage value of horizontal extension

bait=[]; bait(1,1)=X14(2,1); %βangle

for i=2:r

sum=sum+X9(i,1);

if (sum>=20)

sum=0;

k=k+1;

L(k,1)=X10(i,1);

bait(k,1)=X14(i,1);

end

end

xlswrite([out_path ' circumferential analysis1.xlsx'],bait,j);

end
